# High-Resolution Spectral Domain-Optical Coherence Tomography in Multiple Sclerosis, Part II – the Total Macular Volume. The First Follow-Up Study over 2 Years

**DOI:** 10.3389/fneur.2014.00020

**Published:** 2014-02-24

**Authors:** Nermin Serbecic, Fahmy Aboul-Enein, Sven C. Beutelspacher, Adnan Khan, Clemens Vass, Wolfgang Kristoferitsch, Andreas Reitner, Ursula Schmidt-Erfurth

**Affiliations:** ^1^Department of Ophthalmology, Medical University of Vienna, Vienna, Austria; ^2^Department of Ophthalmology, Faculty of Medicine Mannheim, University of Heidelberg, Mannheim, Germany; ^3^Department of Neurology, SMZ-Ost Donauspital, Vienna, Austria; ^4^Nuffield Department of Surgical Sciences, Division of Medical Sciences, University of Oxford, Oxford, UK

**Keywords:** MS, OCT, macula lutea, neurodegenerative diseases, MRI

## Abstract

**Background:** Recent studies investigating the use of optical coherence tomography (OCT) in multiple sclerosis (MS) patients have resulted in wide-ranging and often contradictory outcomes. This is mainly due to the complex etiology and heterogeneity of MS, physiological variations in the retinal nerve fiber layer (RNFL) and/or total macular volume (TMV), and limitations in methodology. It remains to be discovered whether any retinal changes in MS develop continuously or in a stepwise fashion, and whether these changes occur in all or a subset of patients. High-resolution spectral domain-OCT devices (SD-OCT) would be required to detect subtle retinal changes and longitudinal studies would have to be carried out to investigate retinal changes over time. In addition, if the hypothesis is correct, then retinal and global brain tissue changes should be detected in a substantial majority of MS patients and detection should be possible with a high degree of disease activity and/or long disease course.

**Methodology:** In order to address the factors above, 37 MS patients (relapsing–remitting, *n* = 27; secondary progressive, *n* = 10) were examined prospectively on two occasions with a median interval of 22.4 ± 0.5 months [range 19–27]. SD-OCT was utilized with the Spectralis 3.5 mm circle scan protocol (with locked reference images and eye-tracking mode). None of the patients had optic neuritis 12 months prior to study entry or during the observation period.

**Principal Findings:** The initial TMV pattern differed between study participants, but remained relatively unchanged over the 2-year observation period despite high disease activity or long disease course. The TMV correlated well with the RNFL.

**Conclusion:** The significance of differences in TMV (and RNFL) between study participants remains unclear. Until these differences have been explored further, OCT data in MS patients should be interpreted with caution.

## Introduction

It has recently been described that the retinal nerve fiber layer (RNFL) thickness in multiple sclerosis (MS) patients can be reliably visualized by optical coherence tomography (OCT), and that RNFL reduction correlates to diffuse axonal and/or neuronal degeneration throughout the CNS. This is regardless of whether the eyes have previously been affected by optic neuritis (ON). In addition to the ganglion cell and RNFLs, the total macular volume (TMV) has also been described as a reliable variable in the use of OCT in MS patients.

Loss of TMV was described as an additional neuromarker for the degree of global degeneration of myelin, axons, oligodendrocytes, and astrocytes throughout the CNS of MS patients. Furthermore, changes in TMV were suggested to be more reliable at detecting global neurodegeneration than the observed decrease in the RNFL thickness (1–3).

However, some crucial questions still remain:

(1) How and why would 2.5 million axons of both optic nerves be representative of billions of axons within the entire CNS?
(2) Is there a continuous, degenerative process in the entire CNS taking place in every MS patient, and if so, does it proceed at the same pace in all CNS areas simultaneously? How does this hypothesis apply to those MS patients who follow a clear relapsing–remitting disease course with only few relapses and who are not disabled over decades (4)?
(3) The term “neurodegeneration,” when applied to MS, needs a clear definition. What is meant by primary or secondary degeneration of neurons? Neuropathologists still classify MS as a white matter disease, although it has been established for more than a century that myelin, oligodendrocytes, astroglia, and axons are damaged in MS lesions, and that white and gray matter are both affected (5). It seems that, in recent years, there has been significant distortion in semantics, which has impeded uniformity between studies (ranging from the use of *axonal damage* over *degeneration of axons*, or moving from *neuronal degeneration* to “*neurodegeneration*”). In any case, the term “*neurodegeneration*” implies a primary degenerative process of neurons, and thus should be avoided as long as direct scientific evidence is lacking.
(4) Can the hypothesized retinal changes described above be detected with every OCT device, or is there a minimal technical standard required, as the latest high-resolution SD-OCT is currently assumed to be the golden standard. (1, 6–8)?
(5) Can very subtle changes, described above, be separated from gross changes caused by ON or even physiological variations (6–8)?
(6) Are OCT devices really suitable for the detection and monitoring of expected, subtle RNFL- and TMV changes as there is yet no reliable and normative database available for this specific indication (6, 8).

Most importantly, the existing literature and prospective reports utilizing SD-OCT in MS studies must be interpreted with caution, as even physiological variations of the RNFL or TMV (in addition to the hypothesized subtle retinal changes of <2–4 μm/year) were found to exceed the detection-threshold of high-resolution spectral domain-OCT (SD-OCT) devices (8). To date, most of the OCT studies were performed with conventional time-domain OCT, which has proved to be far inferior to high-resolution SD-OCT, lacking the ability to measure changes in RNFLT of identical retinal locations over time (9).

To address the factors described and avoid methodological limitations, we utilized a high-resolution SD-OCT and a well-classified cohort of 27 RRMS and 10 SPMS patients with partly intense disease activity and high relapse-rates over a prolonged observation period of approximately 22.4 ± 0.5 months.

## Materials and Methods

### Participants

This study was approved by the local Ethics Committee (Commission of Medical Ethics of Vienna; Ethic Approval/Registration Number: EK-08-028-0308 and Ethical Commission of the Medical University of Vienna; Ethic Approval/Registration Number: 414/2008). Informed written consent was obtained from all patients and volunteers before study entry.

We recently reported both the demographic and clinical data, the RNFL baseline characteristics of a total of 59 MS (42 RRMS, 17 SPMS) patients, and the follow-up RNFL characteristics of those 37 MS patients (27 RRMS, 10 SPMS) who were willing to participate further (6, 8). The TMV was determined in each case in the same session as the RNFL. Patients were encouraged to drink sufficiently (1–2 L) 1–2 h before each session to guarantee hydration.

In each case, the diagnosis of MS was based on a combination of clinical course, MRI, cerebrospinal fluid (CSF) analysis, and the exclusion of other disorders or diseases (10–12). Oligoclonal bands were found in the CSF samples of all MS patients. Patients with other diseases that reduce RNFL thickness such as glaucoma, anterior ischemic optic neuropathy, high myopia, and congenital abnormalities of the optic nerves were excluded from the study.

Baseline clinical neurological examinations, visual evoked potentials (VEP), and ophthalmologic examinations were performed within 7 days. None of the patients had a history of ON within 12 months prior to the onset of the study. A summary of detailed demographic and clinical data for each patient is given in Table 1 (8).

**Table 1:** **Summary of demographic and clinical data**.

No	MS subtype	Sex	Age at onset	Before first OCT examination					Follow-up		
				Therapy	Relapses*	ON		Age at baseline	Age at follow-up	Relapses*	Therapy
						Right	Left	
1	RRMS	F	34.5	MITOX, GLAT, IFN(b), IFN(a)	7	0	0	40.5	42.25	3	Natalizumab
2	RRMS	F	18.5	IFN(a), IFN(b)	4	0	0	23.5	25.25	3	Natalizumab
3	RRMS	F	36.0	MITOX^1^, IFN(a)	7	0	0	42.0	43.5	2	Natalizumab
4	RRMS	F	31.5	None	3	0	0	38.0	40.25	0	None
5	RRMS	M	40.0	IFN(a)	3	0	0	45.5	47.5	0	IFN(a)^2^; none
6	RRMS	F	28.5	IFN(b)	3	0	0	39.0	40.75	2	IFN(b), natalizumab^3^
7	RRMS	F	43.0	GLAT, IFN(b), none^4^	4	0	0	48.0	49.75	3	None, natalizumab
8	RRMS	F	40.0	None	2	0	0	42.25	44.0	0	None
9	RRMS	M	24.0	None	2	0	0	25.0	26.5	0	None
10	RRMS	F	18.0	GLAT, none^5^	2	0	0	19.75	21.5	1	None
11	RRMS	F	29.75	IFN(a)^6^, none	4	0	0	36.0	37.5	1	None
12	RRMS	M	31.0	IFN(b)	2	0	0	33.25	35.0	1	IFN(b)
13	RRMS	M	51.0	IFN(b)	2	0	0	52.0	54.75	0	IFN(b)
14	RRMS	F	23.75	None	2	0	0	27.0	28.5	0	None^7^
15	RRMS	M	27.5	GLAT	4	0	0	39.0	40.75	0	GLAT
16	RRMS	F	30.0	IFN(b)^8^, none	4	0	0	46.0	48.5	0	None
17	RRMS	M	39.0	IFN(c)	4	0	0	45.0	47.25	1	IFN(c)
18	RRMS	F	20.0	IFN(a)^9^, none	2	0	0	23.0	25.25	2	None
19	RRMS	F	16.0	GLAT	4	0	0	61.0	63.25	0	GLAT
20	RRMS	F	20.5	IFN(b)^10^, none	5	0	0	28.0	30.25	1	None
21	RRMS	F	26.0	IFN(a), IFN(b), MITOX^11^, none	9	1	1	32.0	34.0	0	None
22	RRMS	F	17.75	IFN(a), IFN(b)	6	1	3	19.75	21.75	1	IFN(b)^12^, natalizumab
23	RRMS	F	31.0	IFN(a), IFN(b)	4	1	0	36.0	38.25	0	IFN(b)
24	RRMS	F	20.0	IFN(b)	8	1	1	47.5	49.0	1	IFN(b)
25	RRMS	M	22.5	GLAT, IFN(a), IFN(b), natalizumab	10	0	1	42.5	44.0	0	Natalizumab
26	RRMS	F	20.0	IFN(a)	3	0	4	41.0	42.5	0	IFN(a)
27	RRMS	M	31.0	GLAT^13^, none	2	0	1	33.0	35.25	0	None
28	SPMS	M	40.0	GLAT, MITOX^14^, none	3	0	0	46.5	48.5	0	None
29	SPMS	F	13.0	IFN(b), MITOX^15^, none	5	0	0	27.0	29.25	2	None
30	SPMS	F	38.0	GLAT, none	3	0	0	45.0	47.25	0	None
31	SPMS	F	33.5	None	3	0	0	56.0	58.25	0	None
32	SPMS	M	28.0	IFN(a), IFN(b)	11	0	0	47.25	49.0	1	IFN(b)
33	SPMS	M	25.0	IFN(c), GLAT, IFN(a), IFN(b)	10	1	1	47.5	49.75	1	IFN(b)
34	SPMS	M	22.0	IFN(b)	5	1	0	30.5	32.25	2	IFN(b)^16^, none
35	SPMS	F	16.0	IFN(a), MITOX^17^, none	6	0	2	44.25	46.5	2	None
36	SPMS	F	50.0	GLAT	4	0	2	53.5	55.75	2	GLAT
37	SPMS	M	29.0	IFN(b)	3	0	1	52.0	53.75	0	IFN(b)

ON, optic neuritis; *, relapses treated with high dose steroid pulse therapy; no included patient had an ON within 12 months prior to the beginning of the study; GLAT, glatiramer-acetate 20 mg subcutaneous once daily; MITOX, mitoxantrone; IFN(a), interferon beta 1a intramuscularly once/week; IFN(b), interferon beta 1a (44 μg) subcutaneous trice/week; IFN(c), interferon beta 1b (250 μg) subcutaneous alternate day; 1, discontinued (48 mg mitoxantrone/m^2^ body surface); none, neither specific immunomodulatory or immunosuppressive therapy, drug holiday; 2, drug withdrawal 9 months after first OCT examination; 3, start 7 months before second OCT examination; 4, drug withdrawal 12 months before first OCT examination; 5, drug withdrawal 6 months before first OCT examination; 6, drug withdrawal 20 months before first OCT examination; 7, strict vegan diet caused vitamin B12 and folate deficiency; 8, high titers of anti-interferon autoantibodies, drug withdrawal 14 months before first OCT examination; 9, drug withdrawal 25 months before first OCT examination; 10, drug withdrawal 8 months before first OCT examination; 11, mitoxantrone cumulative dose 96 mg/m^2^ body surface, drug withdrawal 10 months before first OCT examination; 12, change to natalizumab 2 months after first OCT examination; 13, drug withdrawal 6 months before first OCT examination; 14, mitoxantrone cumulative dose 92 mg/m^2^ body surface, drug withdrawal 10 months before first OCT examination; 15, mitoxantrone cumulative dose 92 mg/m^2^ body surface, drug withdrawal 26 months before first OCT examination; 16, drug withdrawal 5 months after first OCT examination 17, mitoxantrone cumulative dose 108 mg/m^2^ body surface, drug withdrawal 27 months before first OCT examination. Table 1 was already published in Ref. (7).

### High-resolution spectral domain-OCT

We used a high-resolution SD-OCT, which combines OCT technology with a confocal scanning laser ophthalmoscope (Heidelberg Engineering, Heidelberg, Germany, Spectralis software version 4.0.3.0, Eye Explorer Software 1.6.1.0, in addition to Eye Explorer software version 1.6.2.0 due to changes by the manufacturer during the follow-up period). A special eye-tracking mode (TrueTrac™) combined with a high scanning speed (40,000 A-scans/s) with an axial resolution of 7 μm and a transversal resolution of 14 μm allows for the reduction of artifacts due to eye movement. Each macular OCT scan is registered and locked to a reference infrared image. In addition, an automatic real-time averaging mode (ART mode) allows for adjustment of the recorded frames to obtain averaged B-scans, which enhances image quality by optimizing the signal-to-noise ratio. The eye tracker and automatic real-time averaging modes of the Spectralis SD-OCT system were used throughout the study. For follow-up scanning, the OCT software could identify previous scan locations and “guide” the OCT laser beam to scan the same location repeatedly. All automated measurements of macular thickness and volume were performed through dilated pupils with a high-resolution macular scan protocol allowing for a more detailed differentiation of retinal layers. (The TMV compounds of inner limiting membrane, nerve fiber layer, ganglion cell layer, inner plexiform layer, inner nuclear layer, outer plexiform layer, outer nuclear layer, external limiting membrane, photoreceptor layer, and retinal pigment epithelium.)

All macular scans were performed by one skilled and trained observer (NS) within one session and repeated if necessary, until a high-quality macular scan was achieved for use in further analysis. Final analysis was only performed on scans without segmentation errors and no manual correction was performed in any case. The observer had no knowledge of any clinical data or the specific baseline data. The SD-OCT imaging protocol comprised 49 B-scans per volume scan of 20° × 20°, and each scan was averaged with 9 frames per B-scan. Topographic macular surface maps were constructed automatically by the OCT software and displayed with numeric averages of the mean thickness for each of the nine map sectors (F, foveal; TI, inner temporal; TO, outer temporal; II, inner inferior; IO, outer inferior; NI, inner nasal; NO, outer nasal; SI, inner superior; SO, outer superior) within three concentric regions of 1, 3, and 6 mm diameter, respectively, as defined by the Early Treatment Diabetic Retinopathy Study (ETDRS).

### Visual function testing, visual field analysis, visual evoked potentials

Visual function testing, visual field analysis, and VEP have been described previously (6, 7).

### Statistics

The statistics used in this study has been described previously (6–8).

## Results

**Figure 1:**
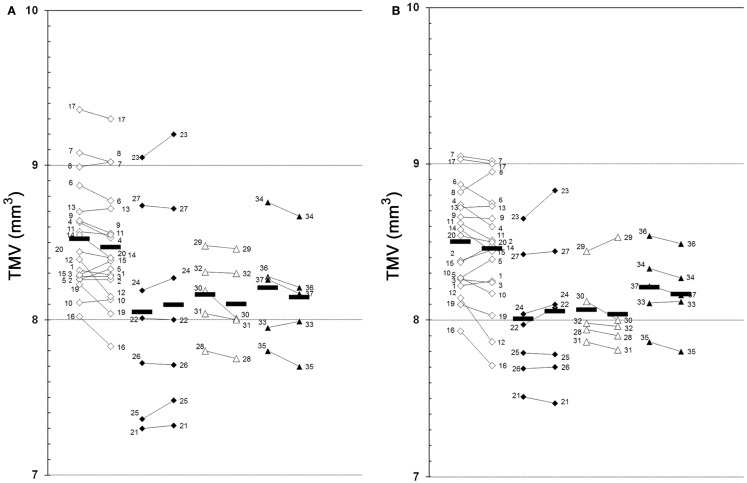
**Global TMV changes between baseline and follow-up examination**. **(A)**, left eye; **(B)**, right eye; 1–37, patient 1–37 (see Table 1 for demographic and clinical data); white squares, RRMS without ON (baseline 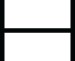 follow-up); black squares, RRMS with ON (baseline 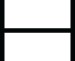 follow-up); white triangles, SPMS without ON (baseline 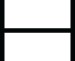 follow-up); black triangles, SPMS with ON (baseline 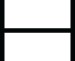 follow-up); black bars, means.

**Figure 2:**
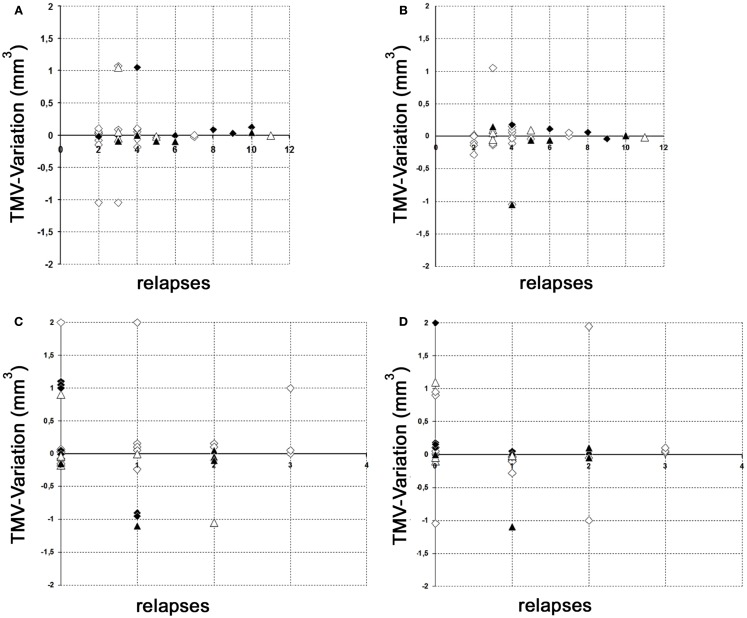
**Variation of global TMV measurements**. **(A,B)** Variation of RNFL measurements between baseline and follow-up examination in relation to the total relapses (without ON) before study entry. **(C,D)** Variation of RNFL measurements between baseline and follow-up examination in relation to the relapses (without ON) during the observation period. **(A)** Left eye; **(B)** right eye; **(C)** left eye; **(D)** right eye; white squares, RRMS without ON; black squares, RRMS with ON; white triangles, SPMS without ON; black triangles, SPMS with ON.

**Figure 3:**
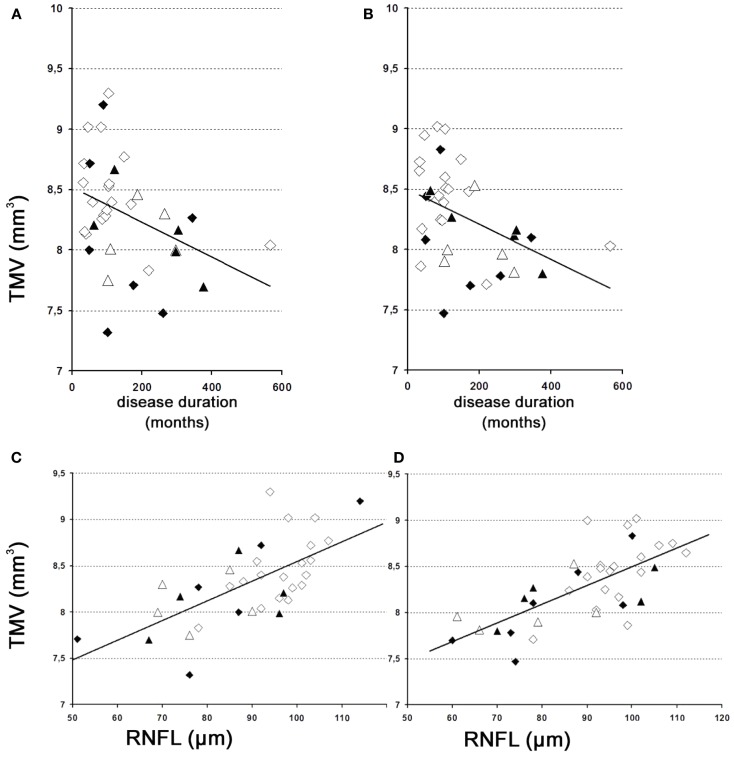
**TMV over time**. **(A,B)** Changes of the TMV over time. **(A)** Left eye – weak correlation of global TMV changes over time. *y* = 8.5 − 0.001 × *x* [correlation coefficient = −0.4; *R*^2^ = 18.7%; standard error of estimate = 0.4]. **(B)** Right eye – weak correlation of global TMV changes over time. *y* = 8.5 − 0.001 × *x* [correlation coefficient = −0.4; *R*^2^ = 14.4%; standard error of estimate = 0.4]. **(C,D)** Correlations between TMV and the RNFL. **(C)** Left eye – moderately strong relationship between TMV and RNFL. *y* = 6.4 + 0.02 × *x* [correlation coefficient = 0.7; *R*^2^ = 47.9%; standard error of estimate = 0.3]. **(D)** Right eye – moderately strong relationship between TMV and RNFL. *y* = 6.4 + 0.02 × *x* [correlation coefficient = 0.7; *R*^2^ = 48.5%; standard error of estimate = 0.3]. **(A)** Left eye; **(B)** right eye; **(C)** left eye; **(D)**, right eye; white squares, RRMS without ON; black squares, RRMS with ON; white triangles, SPMS without ON; black triangles, SPMS with ON.

The results of our study performed with the high-resolution SD-OCT can be summarized briefly as follows: over a median observation period of 22.4 ± 0.5 months (range from 19 to 27 months), the TMV measurements of each MS patient were unchanged compared to baseline (Figure 1). The minimal positive/negative changes seen in some follow-up scans were within the intersession/observer variation (Figures 1 and 2) (3, 6–8). Mean TMV values were found highest in RRMS patients without ON. Mean TMV values were found lower in RRMS with ON and in SPMS with and without ON (Figure 2). The visual and contrast acuity and sensitivity tests (ETDRS, Sloan and Pelli-Robson-charts), the color vision test (Lanthony D-15), the Humphrey visual field analysis, and the VEP also showed no changes compared to baseline values (8). Again, neither age, disease duration, or MS subtype was found to correlate with TMV changes, but TMV values were found moderately correlated to the RNFL values (Figure 3).

## Discussion

A number of cross-sectional OCT-trials have demonstrated a decrease in the RNFLT of patients with MS compared with healthy controls, which was more prominent in progressive disease than in the RR course (13–15). This has led to the assumption that the decrease of RNFLT may reflect “neurodegeneration,” cerebral atrophy, and a progressive disease course (13, 15). This has yet to be confirmed in prospective, controlled longitudinal studies (16). Loss of TMV has been considered as an even more reliable marker for “neurodegeneration” (2).

In this long-term follow-up trial, we were not able to detect any significant reduction of TMV in MS patients over an observation period of 22.4 ± 0.5 months [range 19–27]. All TMV variations (± compared to baseline) were within the intersession and measurement variability of the high-resolution SD-OCT device used in the study (17). The results of our study reflect the negative data of a follow-up investigation on RNFLT in the same cohort of MS patients (6, 8). This is not surprising as axons from the optic nerve originate from receptor ganglion cells of the macula. It should be stated that studies where RNFLT and TMV do not correlate strictly should be interpreted with caution (3). It is likely that if both parameters change (increase or decrease), then they change simultaneously. Which of the two parameters is considered to be more reliable in visualizing the suspected focal (or hypothesized diffuse progressive) subtle changes is still a matter of debate (3) and cannot be answered reliably. To date, the majority of OCT studies were performed to analyze the RNFLT in MS patients. A MEDLINE search reveals 125 results for “MS AND RNFL” but only 6 results for for “MS AND TMV,” e.g., Ref. (1–3, 6–8, 12, 16, 18–20).

Nevertheless, (1) large normative databases and (2) longitudinal studies performed with high-resolution SD-OCT devices, large patient numbers and controls (at least several hundred), and long observation periods over several years (>5 years) are lacking. Meta-analyses are limited as (1) the measurements of individual devices are not interchangeable and comparable (9), (2) inclusion criteria of the patients are not clear, or (3) “affected eyes” were separated from their “unaffected fellow eyes,” hence the case numbers being doubled (20). Most importantly, physiological variations of the RNFLT or TMV were found to exceed the detection-threshold of SD-OCT devices and the hypothesized subtle retinal changes of <2–4 μm/year (7).

Both the current study and our previous studies (6, 8) give rise to a more critical evaluation of OCT as an efficient monitoring instrument for MS disease progression, and thus any interpretation of therapeutic studies must proceed with the utmost caution (1–3, 6–8, 12, 18–21).

## Conflict of Interest Statement

The authors declare that the research was conducted in the absence of any commercial or financial relationships that could be construed as a potential conflict of interest.
