# Corrigendum: “High-Resolution Spectral Domain-Optical Coherence Tomography in Multiple Sclerosis, Part II – The Total Macular Volume. The First Follow-Up Study Over 2 Years”

**DOI:** 10.3389/fneur.2015.00131

**Published:** 2015-06-04

**Authors:** Nermin Serbecic, Fahmy Aboulenein-Djamshidian, Sven C. Beutelspacher, Adnan Khan, Clemens Vass, Wolfgang Kristoferitsch, Andreas Reitner, Ursula Schmidt-Erfurth

**Affiliations:** ^1^Department of Ophthalmology, Medical University of Vienna, Vienna, Austria; ^2^Department of Neurology, SMZ-Ost Donauspital, Vienna, Austria; ^3^Department of Ophthalmology, Faculty of Medicine Mannheim, University of Heidelberg, Mannheim, Germany; ^4^Nuffield Department of Surgical Sciences, Division of Medical Sciences, University of Oxford, Oxford, UK

**Keywords:** multiple sclerosis, optical coherence tomography, retinal nerve fiber layer thickness, total macular volume, spectral domain, time domain, heterogeneity, individual differences

## A Corrigendum on the Affiliation

The affiliations for Wolfgang Kristoferitsch and Fahmy Aboul-Enein is:

Karl Landsteiner Institute for Neuroimmunological and Neurodegenerative Disorders, SMZ-Ost Donauspital, Langobardenstrasse 122, 1220 Wien, Austria

## A Corrigendum on Figure 2

As we confused the terms “RNFL” and “TMV” in the figure legend and submitted the incorrect image for Figure 2, we have to re-submit Figure 2 and correct the figure legend for Figure 2 as follows:

**Figure 2:**
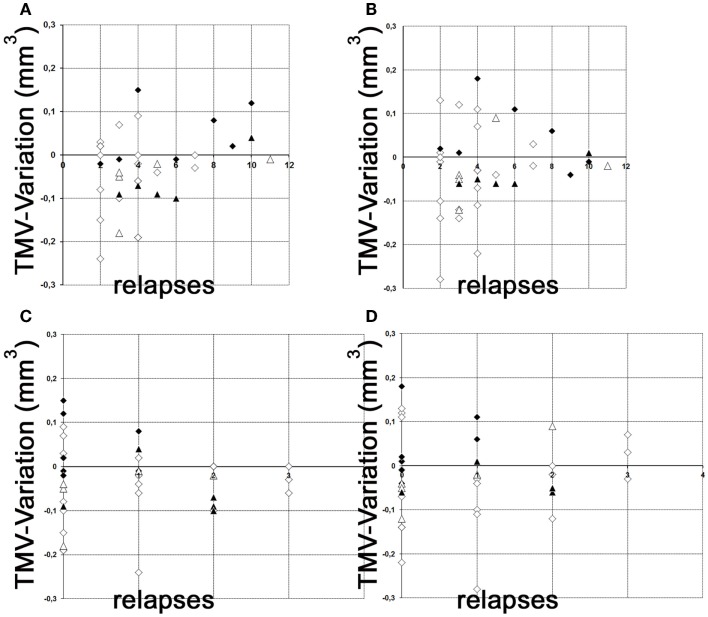
**Variation of global TMV measurements**. **(A,B)** variation of TMV measurements between baseline and follow-up examination in relation to the total relapses (without ON) before study entry. **(C,D)** variation of TMV measurements between baseline and follow-up examination in relation to the relapses (without ON) during the observation period. **(A)** left eye; **(B)** right eye; **(C)** left eye; **(D)** right eye; white squares, RRMS without ON; black squares, RRMS with ON; white triangles, SPMS without ON; black triangles, SPMS with ON. However, neither the raw data nor their interpretations have thus be changed.

## Conflict of Interest Statement

The authors declare that the research was conducted in the absence of any commercial or financial relationships that could be construed as a potential conflict of interest.

